# Cervical spondylotic myelopathy presenting as a “pancake enhancement” on MRI of the spinal cord: A case report and review of literature

**DOI:** 10.1002/ccr3.7052

**Published:** 2023-03-08

**Authors:** Nava Raj Sharma, Bharosa Sharma, Saral Lamichhane, Madalasa Pokhrel, Prajwal Shrestha

**Affiliations:** ^1^ Department of Internal Medicine Manipal College of Medical Sciences Pokhara Nepal; ^2^ Department of Internal Medicine John H. Stroger Jr. Hospital of Cook County Chicago Illinois USA; ^3^ Department of Internal Medicine Gandaki Medical College Pokhara Nepal; ^4^ Department of Internal Medicine Montefiore Medical Center New York City New York USA

**Keywords:** neurology, pancake enhancement, spinal cord, spondylotic myelopathy, upper and lower motor signs

## Abstract

Spondylotic myelopathy involves chronic spinal cord compression from degenerative spine changes presenting a myriad of neurological and pain symptoms. We report a case of cervical myelopathy with transverse pancake‐like gadolinium enhancement seen on MRI in a 42‐year‐old gentleman with progressive bilateral upper extremity numbness, tingling, and impaired gait.

## 1 INTRODUCTION

Spondylotic myelopathy involves chronic compression of the spinal cord from degenerative changes of the spine presenting as a myriad of neurological and pain symptoms. Although it is primarily a clinical diagnosis, the use of advanced imaging, such as magnetic resonance imaging (MRI), allows us to shuffle through various causes of myelopathies rapidly. Transverse pancake‐like gadolinium enhancement is a rare but specific finding on MRI which points toward spondylotic myelopathy.^1^ Recognition of this sign saves time for treatment initiation and is crucial in preventing subsequent disability.

## 2 CASE PRESENTATION

A 42‐year‐old male patient with no significant past medical history presented with approximately 1 year of progressive bilateral upper extremity numbness, tingling, and impaired gait. His symptoms started when he was rear‐ended while driving his car a year prior. Approximately 2 weeks after the car accident, he began to notice numbness and tingling of his fingertips, progressing up his right upper extremity to the shoulder. He then experienced numbness and tingling of his left fingertips, which progressed proximally in a similar pattern. Subsequently, the symptoms involved his bilateral lower extremities. The lower extremity symptoms were associated with difficulty ambulating, especially lifting his left leg off the ground and occasional buckling of both knees causing him to fall. He also endorsed pain at both hips and over his neck musculature but denied pain elsewhere, including his back. With regard to sensation in his hands, only pressure sensation was spared. His only additional complaint was urge urinary incontinence. His slow gait exacerbated this problem. He had no issues with his bowel function.

Physical examination was notable for generalized hypertonia (Left > Right), spasticity, hyperreflexia in all four extremities, positive Hoffman sign in both hands, myoclonus in bilateral ankles, and loss of pinprick sensation in bilateral upper extremities.

**FIGURE 1:**
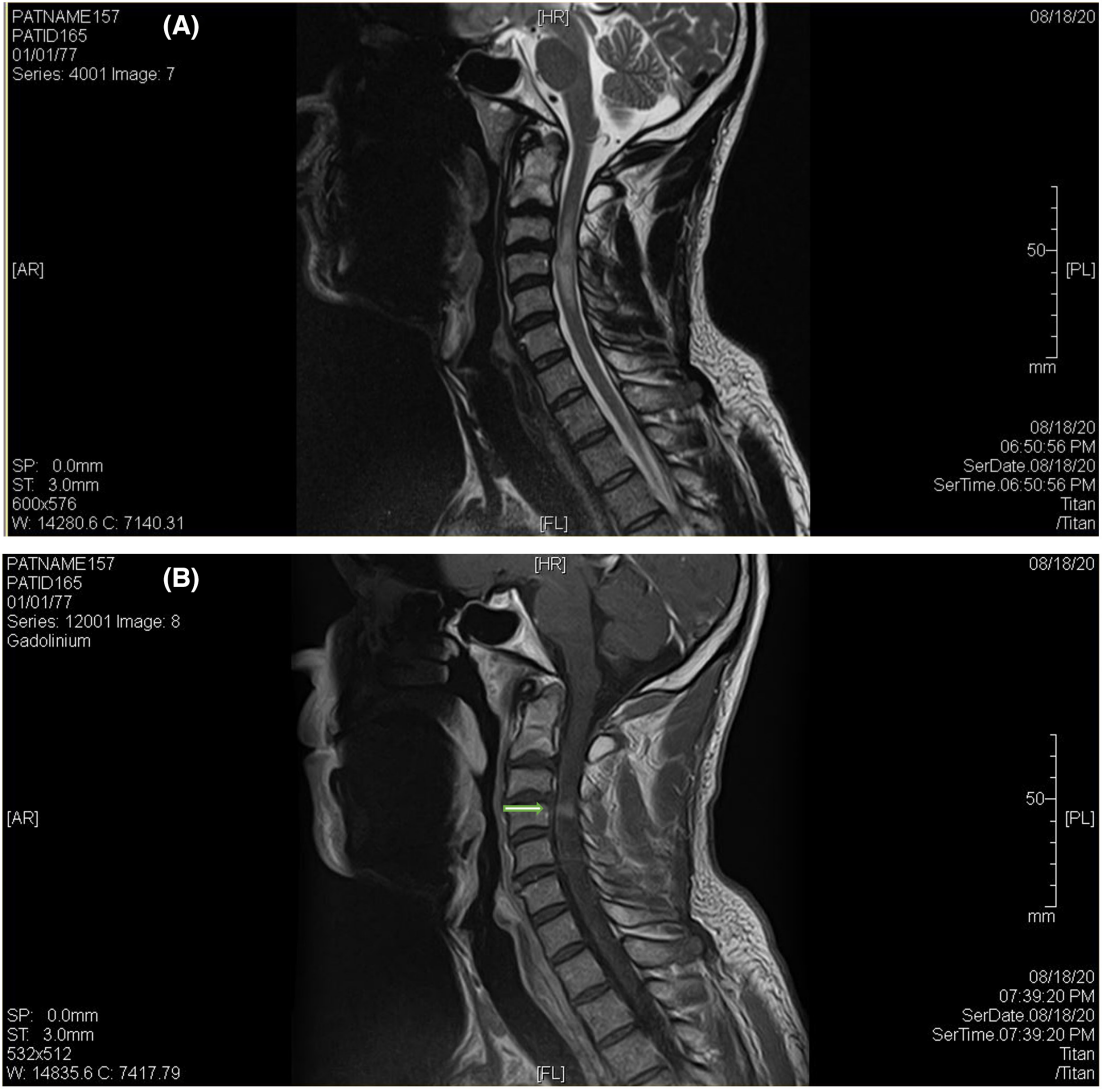
Neuroimaging of spondylosis with gadolinium enhancement. (A) Sagittal magnetic resonance imaging (MRI) reveals T2‐signal hyperintensity in the spinal cord. A long segment of cord edema is seen within the upper spinal cord from the C2–C3 disc space extending to the inferior endplate of C5. A disc bulge at C3–C4 mildly flattens the anterior aspect of the spinal cord. (B) A linear area of contrast with the “pancake enhancement pattern” is seen slightly below the disc level indicated by the arrows.

**FIGURE 2:**
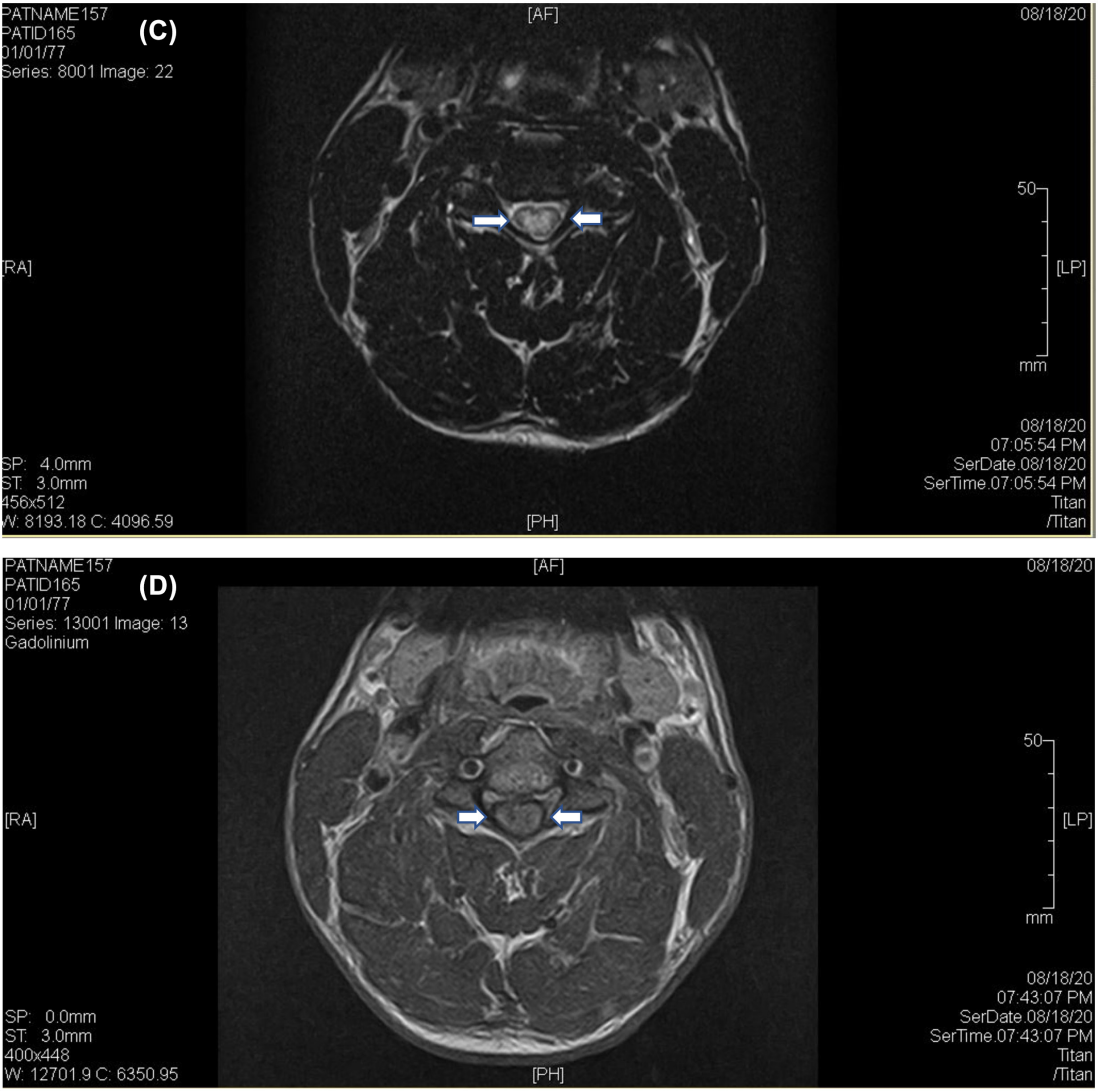
(C, D) Axial gadolinium enhancement in cervical spondylosis.

Tests for human immunodeficiency virus (HIV), thyroid stimulating hormone (TSH), erythrocyte sedimentation rate (ESR), creatine kinase (CK), and syphilis were all unremarkable. Computed tomography (CT) of the cervical spine did not show any fracture or dislocation but did show left‐sided foraminal stenosis at the C3–C4 level. Cervical MRI showed a long cord section of edema and a “pancake enhancement” pattern as illustrated in Figures 1 and 2, corresponding to spondylotic myelopathy. Brain MRI was negative for areas of white matter enhancement, excluding multiple sclerosis and other demyelinating diseases such as acute disseminated encephalomyelitis. Lumbar puncture was not performed as the risks given the cord edema outweighed the benefits.

Hence, due to signs of cord inflammation seen on MRI and the fact that steroids have been tried even in such cases that were compressive triggered pancake sign enhancement,^2^ and that there was no concern for an infective process, the decision was made to start an empiric steroid course with methylprednisolone 1 g daily followed by high dose prednisone and taper. Neurosurgery was also consulted for possible surgical decompression. However, as per their assessment, decompression at the level C3–C4 level would not be beneficial due to the minimal residual compression and the fact that underlying cord enhancement had not been clearly resolved with surgery in previously published literature.^2^ Thus, the patient was planned for a follow‐up MRI of the cervical spine and aggressive rehabilitation to maintain strength.

## 3 DISCUSSION

Myelopathy, encompassing traumatic, vascular, neoplastic, neuropathic, infectious, and any other insult to the spinal cord remain a clinical diagnosis, with specific neurological symptoms and signs that help localize the insult to the spinal cord. Classical upper and lower motor neuron signs are prominent depending on the level of insult. Our patient, having cervical myelopathy, had upper motor neuron (UMN) signs and symptoms in both upper and lower extremities and bladder involvement. Furthermore, a “sensory level” with a band‐like sensation at the superior margin is expected in thoracic and lumbar myelopathies. However, this was absent in our patient. Hoffman's sign was positive, corroborating its frequent association with cervical myelopathy.^3^ The differential diagnosis consists of metabolic, neoplastic, vascular, infectious, and demyelinating conditions. The absence of fever and specific interpretation of imaging findings, including the pattern of enhancement and other features, helps to narrow the differentials. This focal enhancement is mainly caused by the disruption of the blood–brain barrier. Dynamic imaging studies might be one of the keys to showing the cause. Other causes of myelopathy were also investigated and ruled out.^4^

Regarding imaging findings, pancake enhancement of the cervical spine was first reported in 2014 by Flanagan et al.^1^, who reported a similar case of upper extremity weakness and paresthesias. Since then, it has been acknowledged as an important, albeit rare, finding of compressive spondylotic myelopathy. The pancake‐like enhancement of the spinal cord on sagittal imaging and circumferential short segment enhancement in the axial plane is quite distinctive. Its key importance lies in aiding with the distinction between compressive myelopathy and other conditions causing medullary enhancement, including inflammatory myelopathy and intramedullary tumors, which, in the absence of hallmark radiological findings, are often challenging.^5^

## 4 CONCLUSION

The pancake enhancement phenomena on MRI in cervical spondylotic myelopathy is very rare, with only a few other case reports in documented literature. Nonetheless, it is a significant radiological finding that can help provide diagnostic clarity and aid in management, as surgical decompression resulted in partial improvement of neurologic function in one case, albeit with the persistent enhancement of the cord, which has been previously observed.^6^

## AUTHOR CONTRIBUTIONS

**Nava Raj Sharma:** Conceptualization; writing – review and editing. **Bharosa Sharma:** Conceptualization; writing – original draft. **SARAL LAMICHHANE:** Writing – original draft; writing – review and editing. **Madalasa Pokhrel:** Supervision; writing – review and editing. **Prajwal Shrestha:** Resources; writing – review and editing.

## FUNDING INFORMATION

No funding received.

## CONFLICT OF INTEREST STATEMENT

Participating authors have no interests to disclose.

## ETHICAL APPROVAL

None.

## CONSENT

Written informed consent was obtained from the patient to publish this report in accordance with the journal's patient consent policy.

## Data Availability

The data that support the findings of this study are available on request from the corresponding author.
